# Elevated Levels of Plasma Superoxide Dismutases 1 and 2 in Patients with Coronary Artery Disease

**DOI:** 10.1155/2016/3708905

**Published:** 2016-10-17

**Authors:** Ji-Ren Peng, Ting-Ting Lu, Hao-Teng Chang, Xuan Ge, Bian Huang, Wei-Min Li

**Affiliations:** ^1^Department of Cardiology Medicine, Affiliated Dongyang Hospital of Wenzhou Medical University, Dongyang, Zhejiang, China; ^2^Department of Science Education, Affiliated Dongyang Hospital of Wenzhou Medical University, Dongyang, Zhejiang, China; ^3^Graduate Institute of Biomedical Science, China Medical University, Taichung, Taiwan; ^4^Department of Computer Science and Information Engineering, Asia University, Taichung, Taiwan

## Abstract

*Aims*. To measure plasma levels of superoxide dismutases 1, 2, and 3 (SOD1, 2, 3) and determine whether SODs can function as biomarkers for coronary artery disease (CAD).* Patients & Methods*. Patient groups were as follows: patients with stable angina pectoris (SAP, *n* = 33), patients with acute coronary syndrome (ACS, *n* = 49), and controls (*n* = 42). Protein quantification was done using ELISA.* Results*. The concentrations of plasma SOD1 and SOD2 were higher in CAD than in healthy controls. No difference in SOD3 levels between CAD and control groups was found. Limited correlations were found between SODs and gender, age, and severity of coronary artery stenosis.* Conclusions*. Plasma levels of SOD1 and SOD2 were elevated in patients with CAD and might serve as surrogate biomarkers for CAD.

## 1. Introduction

Cardiovascular diseases are the most common cause of death worldwide [1, 2]. In 2010, coronary artery disease (CAD) accounted for one in six deaths in the United States, accounting for 379,559 deaths. It is estimated that 620,000 Americans will suffer a first coronary attack each year and another 295,000 will have a repeat attack [1]. In China, approximately 290 million people had cardiovascular disease in 2010 [3]. Cardiovascular disease in China accounts for 3.5 million deaths each year, accounting for 41% of the total number of deaths per year, and this number is continuously increasing [3]. Currently, 94.9 per 100,000 deaths are estimated to be associated with coronary heart disease in urban areas and 71.3 per 100,000 in rural areas. The annual mortality due to CAD increased from 95.3 in 100,000 in 1999 to 103.4 in 100,000 in 2008 [4]. The known general risk factors for CAD are smoking, hypertension, diabetes mellitus, obesity, metabolic syndrome, physical inactivity, and hyperlipidemia [5]. However, these conventional risk factors reflect only 80–90% of CAD cases. Despite significant improvements in the control of these traditional risk factors in the Chinese population, the incidence of CAD remains high, resulting in serious problems such as substantial medical expense, disability, and even death. Thus, the need for new indicators to assess cardiovascular risk is urgent.

Free radical reactions play an important role in the onset and development of many human diseases [6], including diabetes [7], ischemic injury, stroke, and heart disease [8–11]. Previous research suggests that oxidative stress may contribute to the development of CAD [12, 13]. Oxygen free radicals promote low-density lipoprotein peroxidation, increase the number of foam cells, cause vascular endothelial cell injury, and induce expression of proinflammatory cytokines in endothelia. As a result, the blood vessels thicken, ultimately resulting in stenosis of the arteries [14]. Cells possess efficient antioxidant defense systems to protect them from oxidative damage. These are mainly composed of enzymes such as superoxide dismutases (SODs) and glutathione peroxidase (GPX), which scavenge the reactive oxygen species (ROS) produced by cellular metabolism. The process of coronary atherosclerosis is affected by these important enzymes [15]. SOD, which belongs to an enzyme family that is widely found in human cells and body fluids, protects cells against potential cytotoxicity by catalytically scavenging harmful superoxide radicals (O_2_^−^). SOD achieves this by catalyzing the dismutation of highly reactive O_2_^−^ to O_2_ and H_2_O_2_, a less reactive ROS [16]. Early research showed that SOD is an important marker of lipid peroxidation and of the progression of atherosclerosis correlated with oxidative stress [17]. There are three types of SODs. SOD1, the major intracellular SOD, is mainly localized to the cytoplasm but is also found in nuclei, lysosomes, and peroxisomes. SOD1 requires copper/zinc metal ions as cofactors and is present in many kinds of cells [18]. SOD2 is associated with magnesium and is localized to the mitochondrial matrix. SOD3 is a secretory form found in the vascular extracellular space and is also associated with copper/zinc. It is highly expressed in certain tissues, such as heart, lung, and blood vessels [19].

GPX-1 and SOD have both been described as protective factors in preventing cardiovascular events in patients with CAD [12]. However, some studies have placed in question the effect of SOD activity in CAD [13, 20, 21]. Gupta et al. reported that SOD activity decreased with the development of CAD [21]. Pytel et al. reported that SOD activity in patients with CAD decreased by 17% compared with healthy controls [15]. Zengin et al. reported that elevated SOD1 activity was associated with poor prognosis in patients with CAD [13]. However, to date, few reports have measured plasma concentrations of SODs in patients with CAD or attempted to correlate SOD levels with different types of CAD. In this study, we investigated the plasma levels of SODs in patients with stable angina pectoris (SAP), patients with acute coronary syndrome (ACS), and normal control subjects. We aimed to determine whether the plasma levels of SODs can be used as biomarkers for CAD.

## 2. Methods

### 2.1. Subjects

Participants with CAD who underwent coronary angiography at the Department of Cardiology, Dongyang Peoples' Hospital, between November 2014 and November 2015 and had at least one stenosis of ≥50% in a major coronary artery were enrolled in the study. These participants were divided into a SAP group (19 males and 14 females) and an ACS group (27 males and 22 females) according to clinical manifestation. Age-matched healthy controls (23 males and 19 females) were also recruited. The age of the subjects ranged from 44 to 82 years for controls (mean ± SD = 66.6 ± 7.3 years), 44 to 81 years for the SAP group (66.3 ± 8.7 years), and 38 to 85 years for the ACS group (67.6 ± 11.6 years). All participants were Han Chinese. Informed consent was obtained from each participant, and all procedures were approved by the Institutional Review Board of Affiliated Dongyang Hospital of Wenzhou Medical University. Patients with severe infection, surgery, trauma within the previous month, known cancer, or severe liver or renal insufficiency were excluded.

### 2.2. Coronary Angiography

Coronary angiography was performed in all patients using transradial approaches with left and right Judkins catheters. The major coronary vessels included the left main artery (LM), left circumflex artery (LCX), left anterior descending artery (LAD), and right coronary artery (RCA). Two experienced observers quantified the severity of coronary atherosclerosis for each patient using the Gensini score, an assessment for predicting the likelihood of death or cardiovascular events [22]. The severity score is based on the degree of stenosis: ≤25% (score = 1), 26–49% (score = 2), 50–75% (score = 4), 76–90% (score = 8), 91–99% (score = 16), and 100% (score = 32). In addition, each score was adjusted using artery-specific multipliers described by Gensini [22]: LM = ×5; proximal segment of LAD and proximal segment of LCX = ×2.5; middle segment of LAD = ×1.5; distal segment of LAD, the RCA, the posterolateral artery, and the obtuse marginal artery = ×1; and all other areas = ×0.5.

### 2.3. Measurement of Plasma SOD Levels

Venous blood (10 mL) was collected from patients with SAP and ACS the morning after coronary angiography; samples were also collected from controls at the time of enrollment. All participants were free of acute infection and stress at the time of collection. Serum collected using lithium heparin tubes was harvested by centrifugation at 3000 ×g for 10 min, divided into aliquots, and frozen at −80°C until use. Plasma concentrations of SOD1, SOD2, and SOD3 were determined using commercially available ELISA kits (human SOD1, Cloud-Clone, Houston, TX, USA; human SOD2, Abnova, Taipei, Taiwan; and human SOD3, MDBio, Qingdao, China).

### 2.4. Statistical Analysis

All data were analyzed using Prism v5.0 (GraphPad Software, La Jolla, CA, USA). Continuous variables were expressed as the mean ± SD (standard deviation). Plasma SOD levels among the SAP, ACS, and control groups were compared using one-way analysis of variance. The correlation between ages, severity of coronary atherosclerosis, and SOD concentration was assessed using Pearson correlation analysis. Diagnostic accuracies of SOD concentrations were calculated using a receiver operating characteristic (ROC) curve, and diagnosis performance was measured as the area under the ROC curve. *p* values < 0.05 were considered statistically significant.

## 3. Results

### 3.1. Baseline Characteristics

Clinical characteristics of CAD patients and controls are shown in Table 1. The groups were matched for gender, age, body mass index, smoking habit, hypertension, and diabetes. Compared with the control group, the SAP and ACS groups had significantly higher mean concentrations of total cholesterol (TC, by 10.9% and 20.8%, resp.) and lower HDL cholesterol (HDL-C, by 24.8% and 27.2%, resp.). In addition, ACS patients had higher LDL cholesterol (LDL-C, 28.3% increase) and higher Hs-crp (134.7% increase).

### 3.2. Comparison of SOD Levels

**Figure 1:**
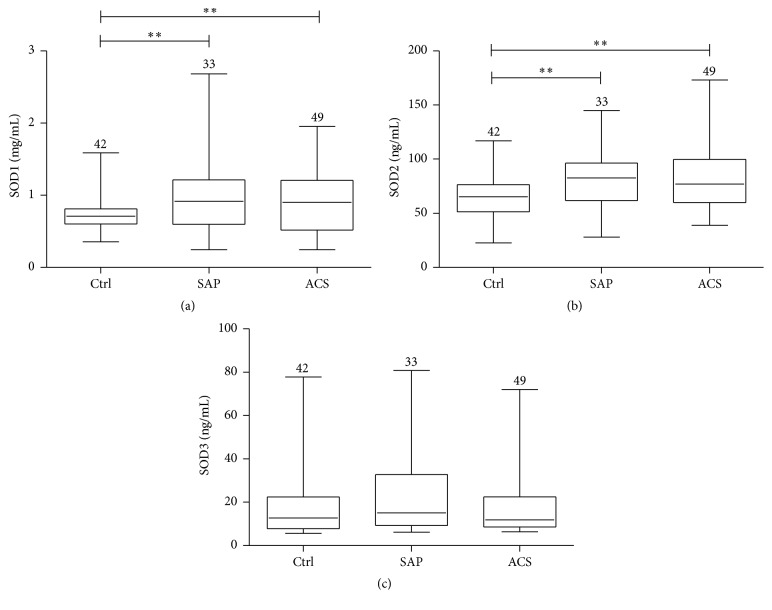
Concentrations of plasma SOD1, SOD2 and, SOD3 in healthy controls (Ctrl), patients with SAP, and patients with ACS. Values for *n* are shown above each error bar. The middle lines mean median values. ^*∗∗*^ *p* < 0.01.

In the control group, the mean concentrations of plasma SOD1 and SOD2 were 0.73 ± 0.22 mg/mL and 64.9 ± 18.5 ng/mL, while the medians were 0.71 mg/mL and 65.2 ng/mL, respectively (Figure 1). Compared with control, SOD1 and SOD2 concentrations in the SAP group were elevated by 37.6% (1.0 ± 0.53 mg/mL, median = 0.92 mg/mL, and *p* = 0.003) and 28.3% (83.2 ± 27.2 ng/mL, median = 82.5 ng/mL, and *p* = 0.001), respectively. Similarly, relative to control, SOD1 and SOD2 concentrations were elevated in the ACS group by 28.1% (0.93 ± 0.45 mg/mL, median = 0.90 mg/mL, and *p* = 0.008) and 30.8% (85.0 ± 34.6 ng/mL, median = 76.9 ng/mL, and *p* = 0.001). The concentrations of these two proteins were not significantly different between the SAP and ACS groups (*p* = 0.53 for SOD1 and *p* = 0.81 for SOD2). For SOD3, there were no significant differences among the SAP, ACS, and control groups (22.6 ± 18.3, 16.1 ± 11.9, and 18.3 ± 15.5 ng/mL, while the medians were 15.1, 11.9, and 12.8 ng/mL, resp.; *p* > 0.05). These results suggest that plasma concentrations of SOD1 and SOD2 but not SOD3 maybe useful as biomarkers for diagnosis of CAD.

### 3.3. Performance of SODs in the Prediction of CAD

**Figure 2:**
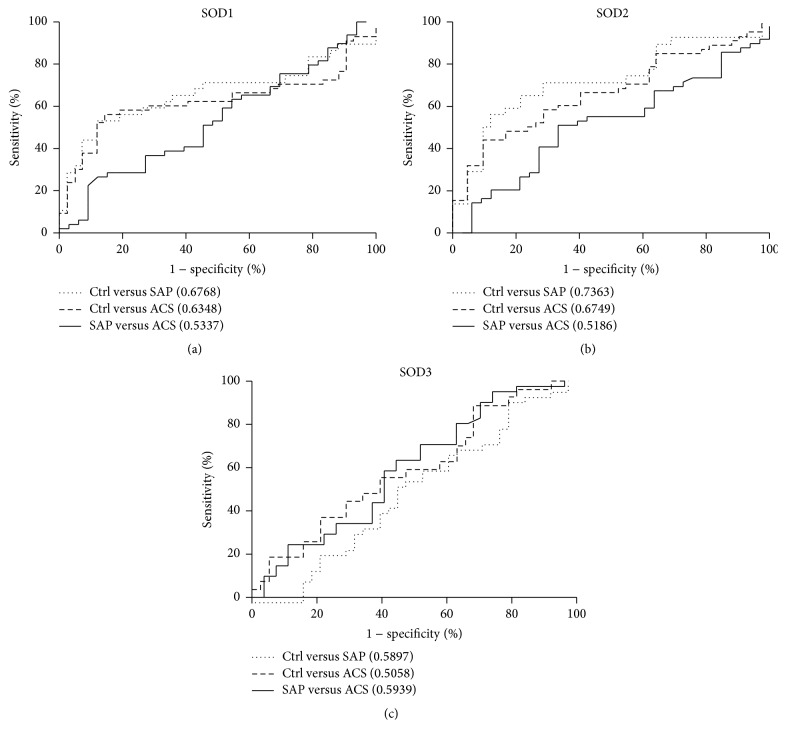
ROC curves of SODs for the diagnosis of CAD. The area under the curve is shown in parentheses for each curve.

We distinguished between patients with SAP or ACS and controls by measuring the area under receiver operating characteristic (ROC) curves. At a cut-off value of 0.8962 mg/mL, SOD1 exhibited the best performance with 54.55% sensitivity and 88.10% specificity and an area under the curve (AUC) of 0.6768 for SAP versus control (Figure 2(a)). In the comparison between ACS and control, SOD1 showed 57.14% sensitivity and 85.71% specificity with an AUC of 0.6348 and a cut-off value of 0.8725 mg/mL. SOD1 showed no difference between the SAP and ACS groups. In the comparison between SAP and control, SOD2 showed 57.58% sensitivity and 88.10% specificity with an AUC of 0.7363 at a cut-off value of 80.49 ng/mL (Figure 2(b)). In the comparison between ACS and control, SOD2 showed 44.90% sensitivity and 90.48% specificity with an AUC of 0.6749 at a cut-off value of 82.80 ng/mL. There was no difference between these two patient groups. SOD3 levels showed no significant differences among the three subject groups (Figure 2(c)). Based on this analysis, the plasma level of SOD2 performed best as a biomarker for the diagnosis of CAD.

### 3.4. Correlation between Severity of Coronary Artery Stenosis and SOD Levels

**Figure 3:**
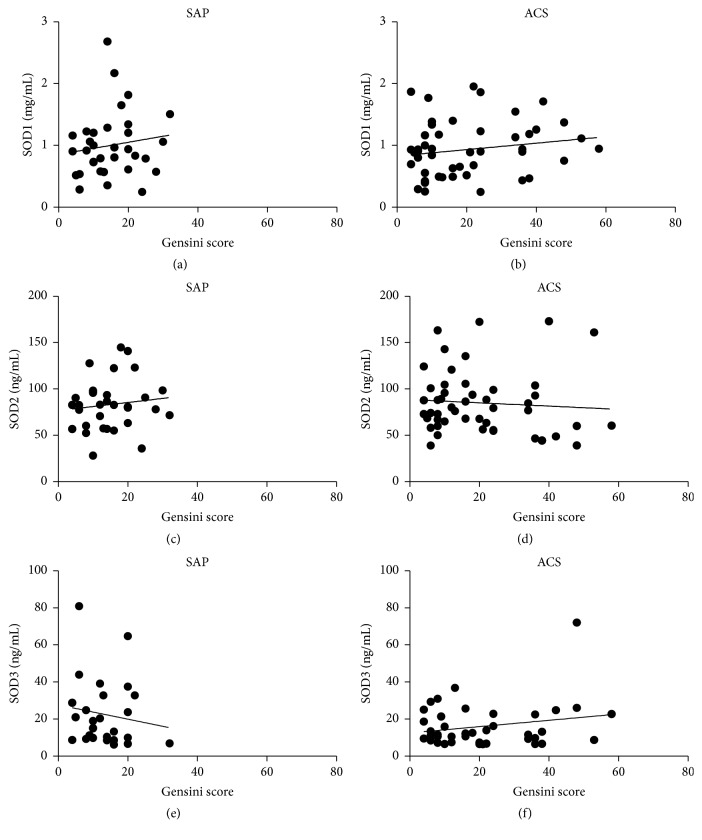
Correlations between Gensini scores and SOD levels in patients with SAP or ACS.

The severity of coronary artery stenosis for each patient with CAD was determined using the Gensini score [22], and the correlation between these scores and plasma SOD levels was analyzed (Figure 3). There was no significant correlation between concentrations of plasma SODs and severity scores in patients with SAP (SOD1: *r* = 0.14 and *p*  = 0.44; SOD2: *r* = 0.12 and *p*  = 0.50; and SOD3: *r* = 0.04 and *p*  = 0.84) or ACS (SOD1: *r* = 0.17 and *p*  = 0.24; SOD2: *r* = 0.07 and *p*  = 0.61; and SOD3: *r* = 0.14 and *p*  = 0.38).

### 3.5. Correlation between SODs and Gender or Age

**Figure 4:**
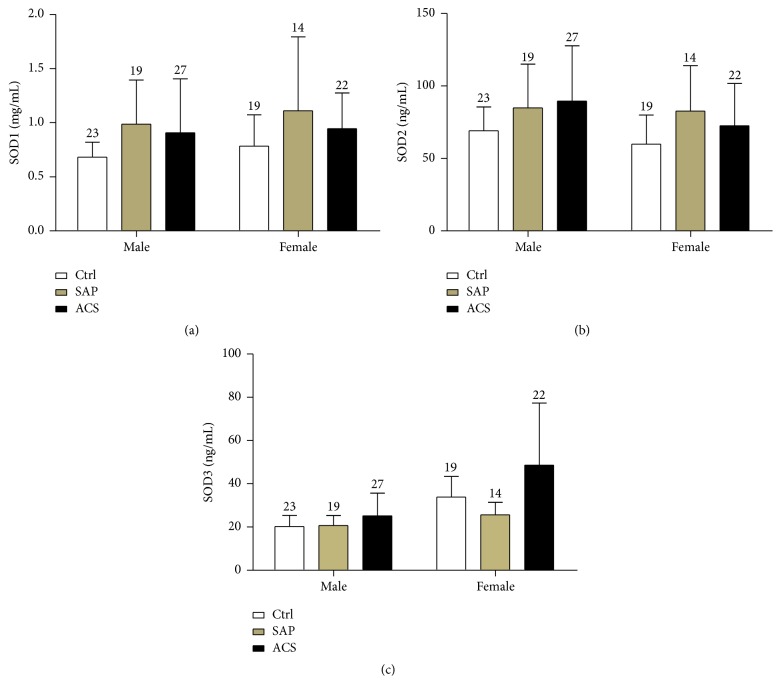
Levels of SODs by gender in controls and patients with SAP or ACS. Values for *n* are shown above each error bar.

**Figure 5:**
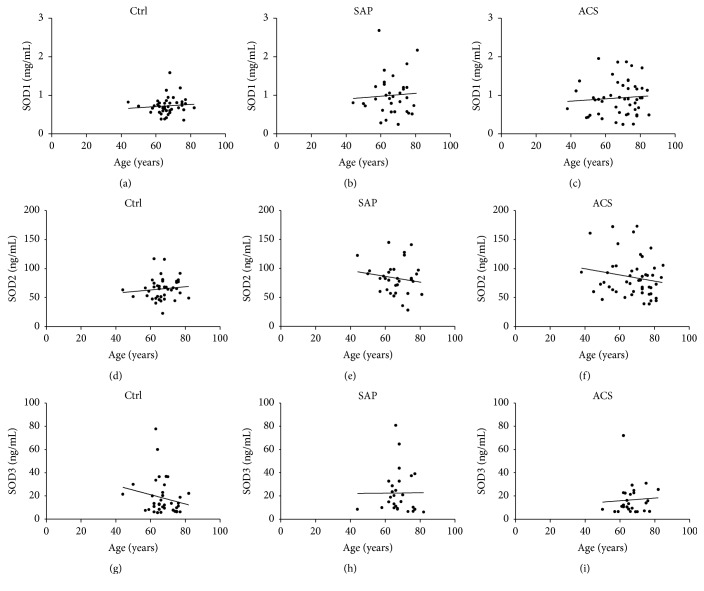
Correlations between age and SOD levels in controls and patients with SAP or ACS.

The concentrations of SOD1 and SOD2 were observed to be significantly higher in male patients with CAD than in male controls (*p* < 0.05; Figures 4(a) and 4(b)). The level of SOD2 in female patients with SAP was significantly higher compared with female controls (*p* < 0.05), but the average levels of SOD1 were not different in females across the three groups. The concentration of SOD3 was not significantly different among the three groups for either gender (Figure 4(c)). In addition, levels of SODs were not significantly correlated with age (Figure 5) among patients with SAP (SOD1: *r* = 0.06 and *p*  = 0.73; SOD2: *r* = 0.16 and *p*  = 0.38; and SOD3: *r* = 0.02 and *p*  = 0.97), patients with ACS (SOD1: *r* = 0.08 and *p* = 0.59; SOD2: *r* = 0.18 and *p*  = 0.22; and SOD3: *r* = 0.25 and *p*  = 0.19), or control subjects (SOD1: *r* = 0.09 and *p*  = 0.56; SOD2: *r* = 0.11 and *p*  = 0.49; and SOD3: *r* = 0.19 and *p*  = 0.25).

## 4. Discussion

SOD catalyzes the dismutation of O_2_^•−^ to H_2_O_2_, which can be reduced to H_2_O by catalase and GPX. By inhibiting the effects of oxidative alterations induced by O_2_^•−^, SOD is believed to prevent atherogenesis and associated cellular responses such as apoptosis, hypertrophy, and cell migration [23–25]. These results indicate the potential protective role of SODs against atherosclerosis. Elevated SOD1 activity confers protection against acute or chronic oxidative injury, including atherosclerosis [26, 27]. Gupta et al. also reported that the enzymatic activity of SOD decreases with the development of CAD [21]. In contrast, a pathological role for SOD1 was reported in which an increase of SOD1 activity enhances oxidative injury by increasing the rate of formation of distal oxidants and elevating H_2_O_2_ to a toxic level [28, 29]. SOD2 can regulate mitochondrial ROS and control endothelial dysfunction and apoptosis, leading to the development of atherosclerosis [30]. However, previous research reported controversial findings for the effect of SOD activity relative to CAD. Gupta et al. and Pytel et al. reported that the activity of SOD decreases in CAD [15, 21], whereas Zengin et al. drew the opposite conclusion [13]. These inconsistencies may be due to the possibility that high SOD activity enhances oxidative injury by increasing the rate of formation of distal oxidants. It has been suggested that the effects of SOD are dose dependent and are characterized by a bell-shaped curve [31, 32]. In the present study, plasma levels of SOD1 and SOD2 in patients with CAD (both SAP and ACS) were higher than those in healthy controls, which is consistent with a previous study [13], assuming that higher plasma SOD levels correlate with higher SOD activity. The regulation of O_2_^•−^ and H_2_O_2_ is complex and depends on both the equilibrium and pathophysiologic changes within a cell. When this balance is broken, multiple biochemical reactions can be affected, potentially resulting in damage to the cell as a result of relatively high SOD activity. Some reports suggest that SOD3 may play a role in atherosclerosis, but its functional significance is unclear. Wang et al. reported that decreasing plasma SOD3 levels are associated with increasing history of myocardial infarction [33]. Sentman et al. concluded that SOD3 might have little effect on the development of atherosclerosis [34]. Consistent with this latter report, we found that the plasma level of SOD3 in patients with CAD was not significantly different from that in control subjects, suggesting that SOD3 might do little in the development of atherosclerosis.

The present study found no significant difference between patients with SAP and those with ACS for plasma levels of any of the three SODs. We found that Gensini scores of patients with CAD were not significantly correlated with plasma levels of SODs, regardless of the kind of CAD (SAP or ACS). A possible explanation for these results is that the increase in SODs may be compensated by a common mechanism, such as oxidative stress [26, 27]. These results suggest that the plasma levels of SODs in CAD patients may not be affected by coronary plaque formation, coronary atherosclerosis rupture, or thrombosis.

We collected subjects with a smoking habit and compare the SOD levels among three groups. SOD1 and SOD2 concentrations in the SAP and ACS group were elevated as compared with control. Similarly, no significant difference of SOD1 and SOD2 was observed between SAP and ACS groups. There was no significant difference of SOD3 among the three groups. To compare the smoking effects, we compare the SOD levels in controls with/without a smoking habit. The mean concentration of plasma SOD1 with nonsmoking was minorly elevated; meanwhile no significant difference for SOD2 and SOD3 levels between groups with or without smoking was found. We also compared SOD levels in subjects with diabetes and hypertension among three groups. The levels of SOD1, SOD2, and SOD3 in the three groups with diabetes were not significantly changed. However, SOD1 and SOD2 concentrations in the SAP and ACS subjects with hypertension were elevated as compared with control. SOD3 showed no difference. Although we cannot conclude the disease effects toward SOD levels, similarly, with overall comparison, the SOD1 and SOD2 were expressed higher in plasma of CAD patients as compared with control in the smoking or hypertension groups and SOD3 showed no significant difference in control, SAP, and ACS groups, excluding the diabetes patients. This issue could be analyzed in the future.

Among the three SODs, SOD2 was most promising as a biomarker for predicting CAD, with an AUC of 0.74 for SAP versus control and 0.67 for ACS versus control. Although this prediction level does not meet current standards for Good Medical Practice, this study provides valuable information about the circulating concentrations of SODs and the potential effects of SODs on CAD.

## 5. Limitation

The following limitations to the study should be considered. First, the measurement of SOD levels was performed at only one time point and thus cannot provide information on changes in SOD levels in individual patients. Second, evaluation of the percentage of coronary artery stenosis was subjective; however, it was assessed independently by two experienced cardiologists. Third, the subjects took some medications, such as atorvastatin, aspirin, clopidogrel, calcium channel blocker, angiotensin converting enzyme inhibitor (ACEI), angiotensin receptor antagonist (ARB), and beta blocker. It is reported that statins, ACEI, ARB, and beta blocker may affect oxidative stress [35–37]. The medication effects to influence the levels of SODs were not considered in the experimental design. Like our analyses of medication use described above, the SOD levels cannot be summed up either.

## 6. Conclusion

Plasma levels of SOD1 and SOD2 were elevated in patients with CAD. These enzyme levels may be useful in the future as biomarkers for diagnosis of CAD.

## Figures and Tables

**Table 1:** Characteristics of coronary artery disease (CAD) patients and controls^a^.

Characteristic^b^	Control (*n* = 42)	SAP (*n* = 33)	ACS (*n* = 49)	*p* values^c^
Age (years)	66.6 ± 7.3	66.3 ± 8.7	67.6 ± 11.6	0.852; 0.627; 0.569
Male, *n* (%)	23 (54.8)	19 (57.6)	27 (55.1)	0.811; 0.974; 0.827
Diabetes, *n* (%)	7 (16.7)	6 (18.2)	8 (16.3)	0.866; 0.966; 0.829
Hypertension, *n* (%)	24 (57.1)	18 (54.5)	30 (61.2)	0.825; 0.697; 0.825
BMI (kg/m^2^)	25.4 ± 2.4	23.7 ± 2.4	23.2 ± 3.6	0.129; 0.159; 0.482
Smoking habit, *n* (%)	23 (54.8)	16 (48.5)	25 (51.0)	0.595; 0.725; 0.482
Hs-crp (mmol/L)	1.21 ± 0.49	1.72 ± 0.96	2.84 ± 2.15	0.104; **0.016**; **0.011**
TC (mmol/L)	4.13 ± 0.89	4.58 ± 1.04	4.99 ± 1.05	**0.040**; **0.001**; 0.117
TG (mmol/L)	1.77 ± 0.80	2.11 ± 1.42	1.63 ± 1.01	0.228; 0.495; 0.079
LDL-C (mmol/L)	2.30 ± 0.82	2.52 ± 0.92	2.95 ± 1.03	0.261; **0.002**; 0.075
HDL-C (mmol/L)	1.31 ± 0.27	1.05 ± 0.22	1.03 ± 0.23	**<0.001**; **<0.001**; 0.70

^a^BMI: body mass index; HDL-C: high-density lipoprotein cholesterol; Hs-crp: high-sensitivity C-reactive protein; LDL-C: low-density lipoprotein cholesterol; TC: total cholesterol; and TG: triglycerides.

^b^Values for age, BMI, Hs-crp, and lipid analysis represent the mean ± SD.

^c^Order of *p* values: SAP versus ctrl; ACS versus ctrl; and SAP versus ACS. Bold indicates significant differences (*p* < 0.05).
